# Supplementary material for: Modifying redox properties and local bonding of Co3O4 by CeO2 enhances oxygen evolution catalysis in acid
Source: Nat Commun. 2021 May 24;12:3036. doi: 10.1038/s41467-021-23390-8 (PMC8144612; doi:10.1038/s41467-021-23390-8)
Supplement: Supplementary file 2 — Supplementary Information [file 41467_2021_23390_MOESM2_ESM.pdf]

Supplementary Information

Modifying redox properties and local bonding of Co_3O_4 by CeO_2 enhances oxygen evolution catalysis in acid

Jinzhen Huang[1,2], Hongyuan Sheng[1], R. Dominic Ross[1], Jiecai Han[2], Xianjie Wang[3], Bo Song*[,2] and Song Jin*[,1]

[1]Department of Chemistry, University of Wisconsin–Madison, 1101 University Avenue, Madison, Wisconsin 53706, United States

[2]Center for Composite Materials and Structures, Harbin Institute of Technology, Harbin 150001, China

[3]Department of Physics, Harbin Institute of Technology, Harbin 150001, China

*Correspondence to: jin@chem.wisc.edu (S. J.); songbo@hit.edu.cn (B. S.)

Table of Contents

Supplementary Figure 1..........S3
Supplementary Figure 2..........S4
Supplementary Figure 3..........S5
Supplementary Figure 4..........S6
Supplementary Figure 5..........S7
Supplementary Figure 6..........S8
Supplementary Figure 7..........S9
Supplementary Figure 8..........S10
Supplementary Figure 9..........S11
Supplementary Figure 10..........S12
Supplementary Figure 11..........S13
Supplementary Figure 12..........S14
Supplementary Figure 13..........S15
Supplementary Figure 14..........S16
Supplementary Figure 15..........S17
Supplementary Figure 16..........S18
Supplementary Figure 17..........S19
Supplementary Figure 18..........S20
Supplementary Figure 19..........S21
Supplementary Figure 20..........S22
Supplementary Figure 21..........S23
Supplementary Figure 22..........S24
Supplementary Figure 23..........S25
Supplementary Figure 24..........S26
Supplementary Figure 25..........S27
Supplementary Figure 26..........S28
Supplementary Figure 27..........S29
Supplementary Figure 28..........S30
Supplementary Figure 29..........S31
Supplementary Figure 30..........S32
Supplementary Figure 31..........S33
Supplementary Table 1..........S34
Supplementary Table 2..........S34
Supplementary Table 3..........S35
References cited in the Supplementary Information..........S36

Supplementary Figures and Tables

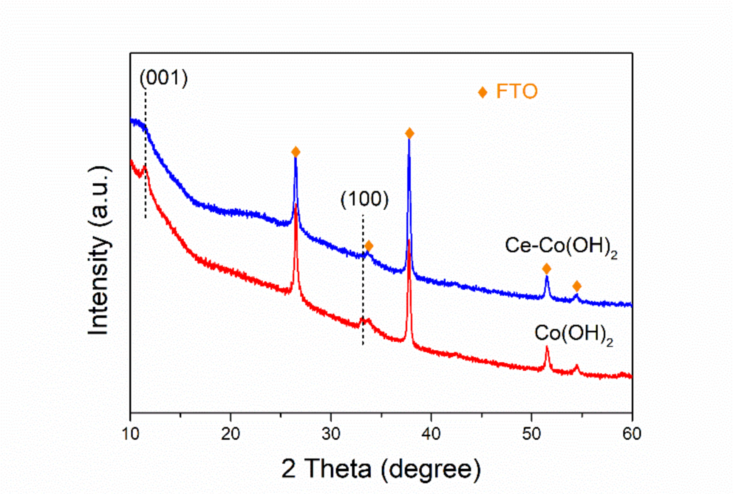

Supplementary Figure 1. PXRD patterns of α-$Co(OH)_2$ and Ce-incorporated $Co(OH)_2$ [denoted as Ce-$Co(OH)_2$] precursors electrodeposited on FTO substrates.

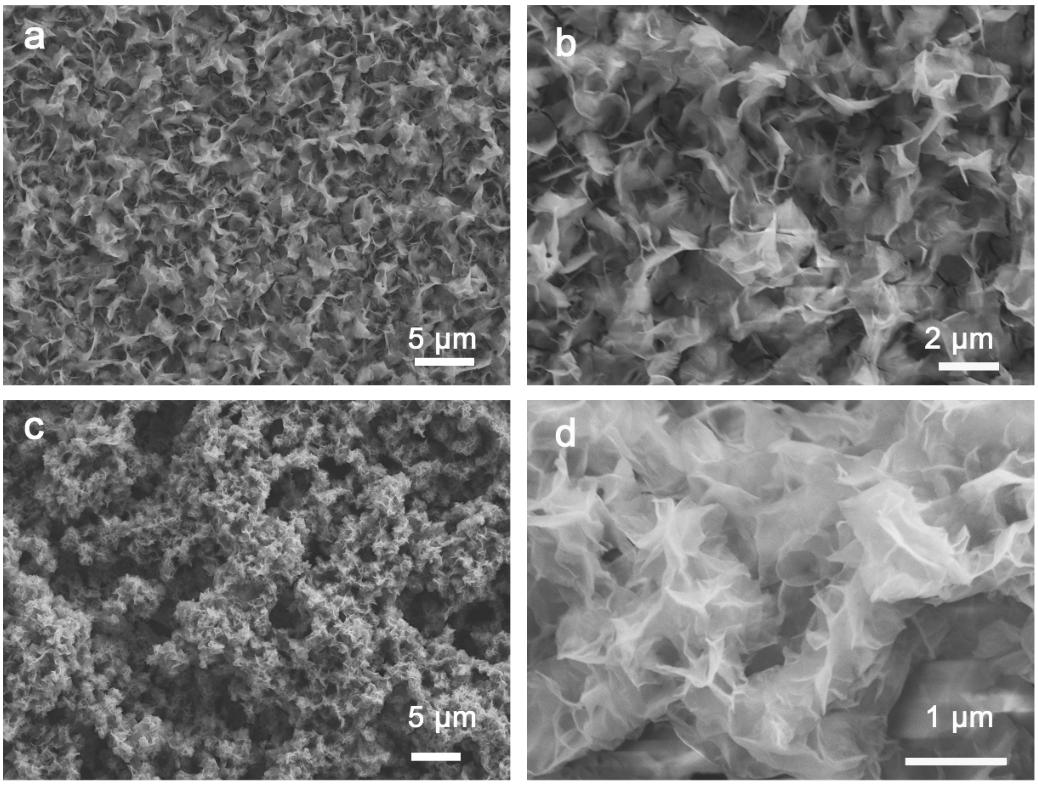

Supplementary Figure 2. Low- and high-magnification SEM images of (a,b) $Co(OH)_2$ and (c,d) Ce-incorporated $Co(OH)_2$ [denoted as Ce-$Co(OH)_2$] precursors electrodeposited on FTO substrates. The $Co(OH)_2$ nanosheets are more uniformly interconnected, while the Ce-$Co(OH)_2$ nanosheets are more aggregated with wrinkles.

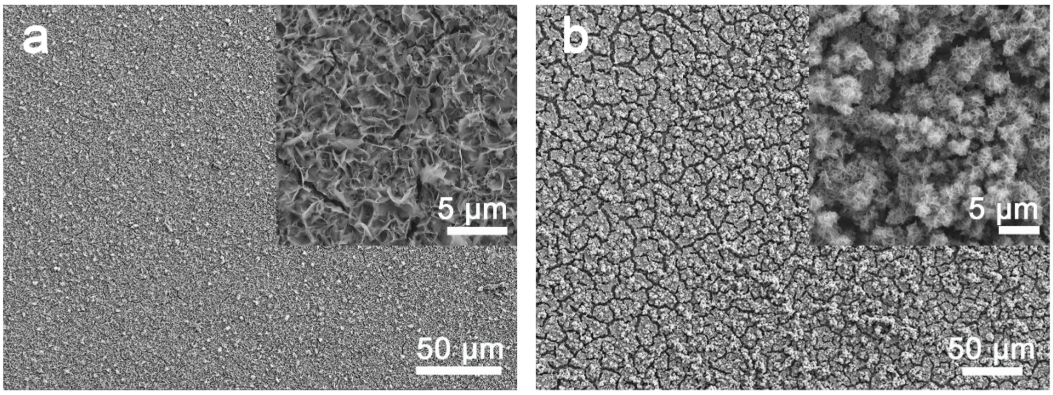

Supplementary Figure 3. Low- and high-magnification SEM images of (a) Co_3O_4 nanostructures and (b) Co_3O_4/CeO_2 nanocomposites on FTO substrates made by annealing the metal hydroxide precursors shown in Supplementary Fig. 2. The annealing process introduced some cracks in both Co_3O_4 and Co_3O_4/CeO_2 films, probably due to the shrinkage during the structural transformation from hydroxides to oxides.

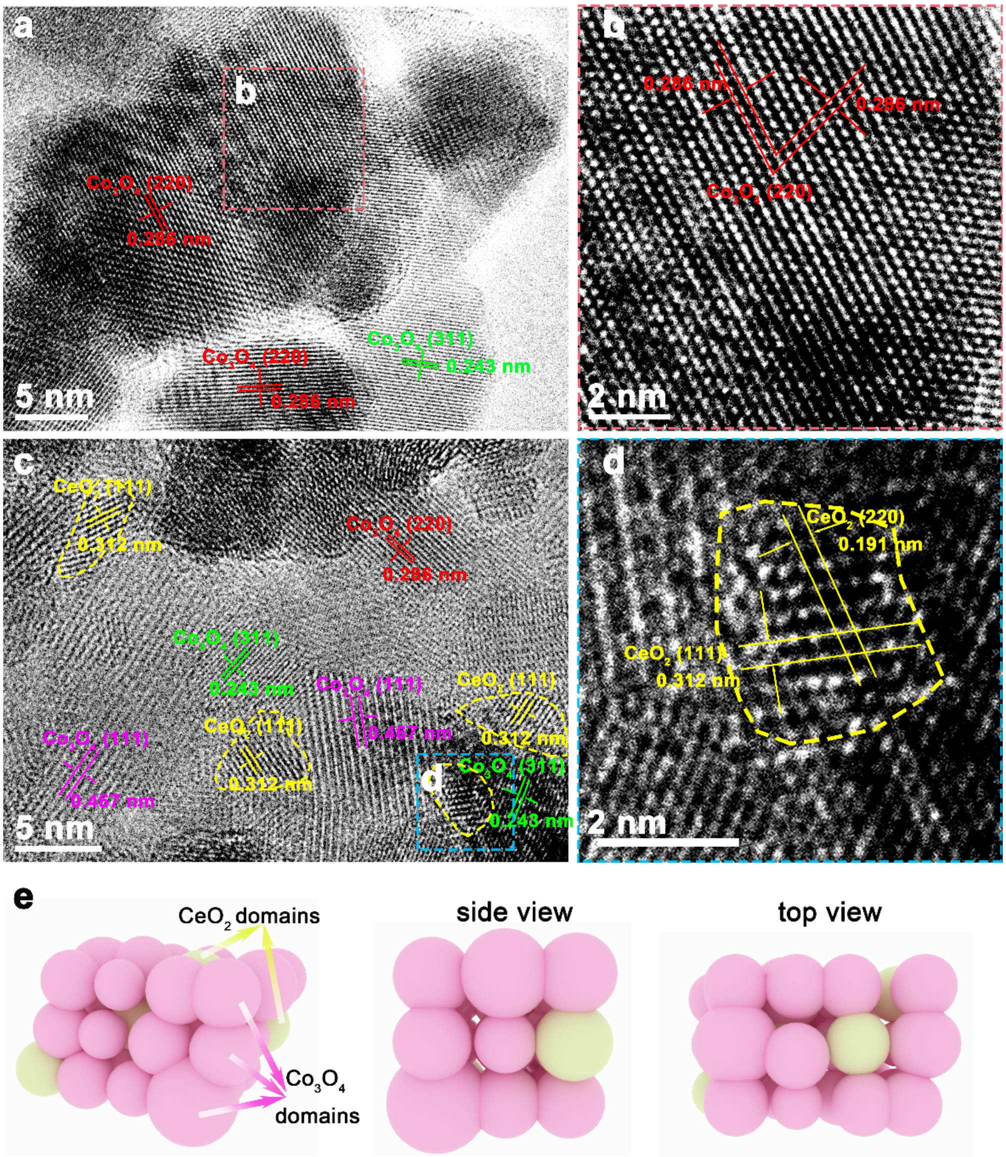

Supplementary Figure 4. HRTEM images of (a,b) Co_3O_4 and (c,d) Co_3O_4/CeO_2 catalysts (see Figure 1a-d in the main text for reference). (b) and (d) are the enlarged images from the region in (a) and (c) highlighted by the dashed boxes, respectively. The CeO_2 domains in (c) are all around 5 nm, which is consistent with calculated results from the PXRD patterns in Supplementary Figure 6. (e) The schematic illustration for the distribution of CeO_2 domains in the Co_3O_4/CeO_2 composite.

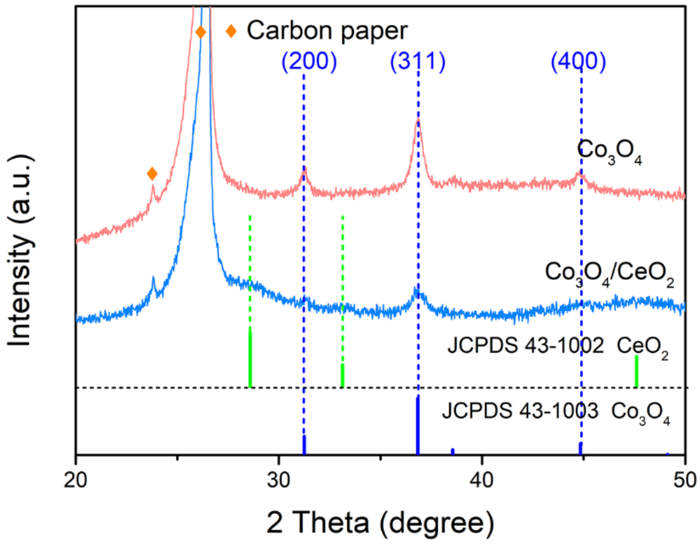

Supplementary Figure 5. PXRD patterns of Co_3O_4 and Co_3O_4/CeO_2 samples grown on carbon paper substrates by annealing at 400 °C for 2 h. Since one of the diffraction peaks of the FTO substrate is overlapped with the (311) diffraction peak of Co_3O_4 (see Fig. 1e in the main text), we synthesized Co_3O_4 and Co_3O_4/CeO_2 samples on carbon paper substrates for the estimation of the average crystalline domain sizes using the Scherrer equation. These were also the catalyst samples grown on high surface area carbon paper electrodes that were electrochemically measured later (Supplementary Fig. 26, 29 and 30).

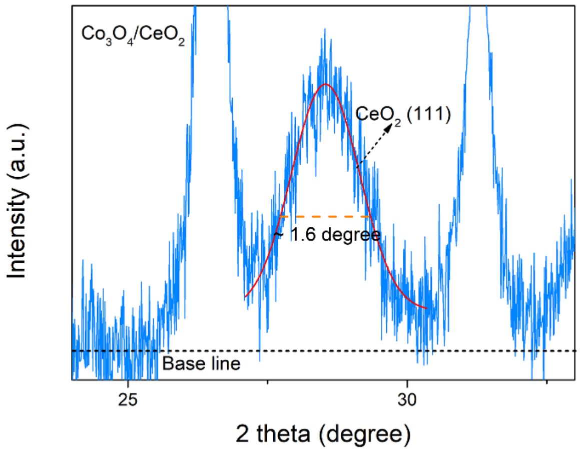

Supplementary Figure 6. The enlarged PXRD pattern of Co_3O_4/CeO_2 at around the CeO_2 (111) peak position. Scherrer analysis of this peak at a full width at half maximum of ~ 1.6 degree reveals the average crystal domain size of CeO_2 is around ~ 5 nm.

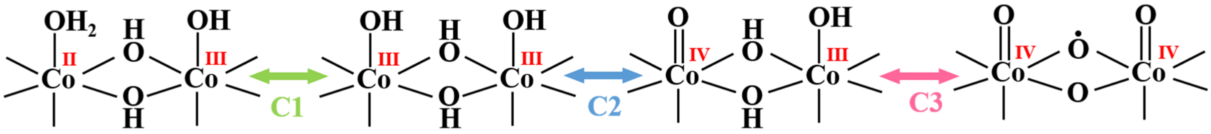

Supplementary Figure 7. Proposed structural motifs associated with the three sets of pre-OER redox features present in the Co_3O_4 catalyst in acidic media that involve dimeric Co redox centers. The corresponding cathodic peaks at ~1.40, ~1.50 and ~1.63 V vs. RHE are denoted as C1, C2, and C3, respectively (see Fig. 2b in the main text). Note that these proposed structural motifs of dimeric Co redox centers are one set of the possible configurations in acidic media, since most of the relevant literature focused on neutral and alkaline media[1-5].

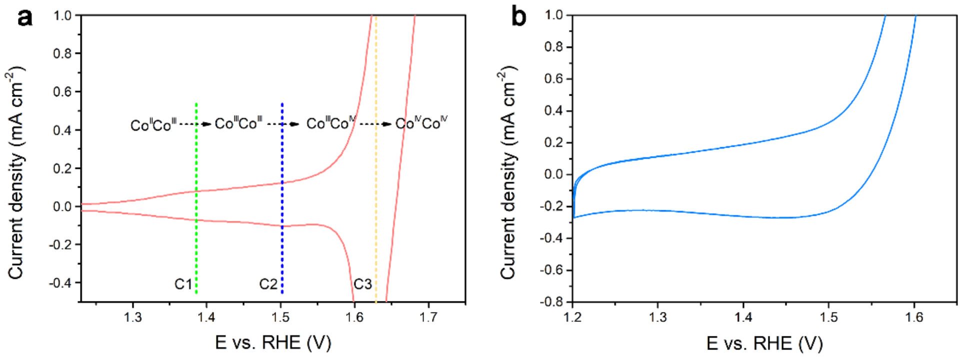

Supplementary Figure 8. Magnified CV curves of the (a) Co_3O_4 and (b) Co_3O_4/CeO_2. The Co_3O_4/CeO_2 nanocomposite catalyst that clearly shows no obvious pre-OER redox features (also see Fig. 2a in the main text for reference full range CV).

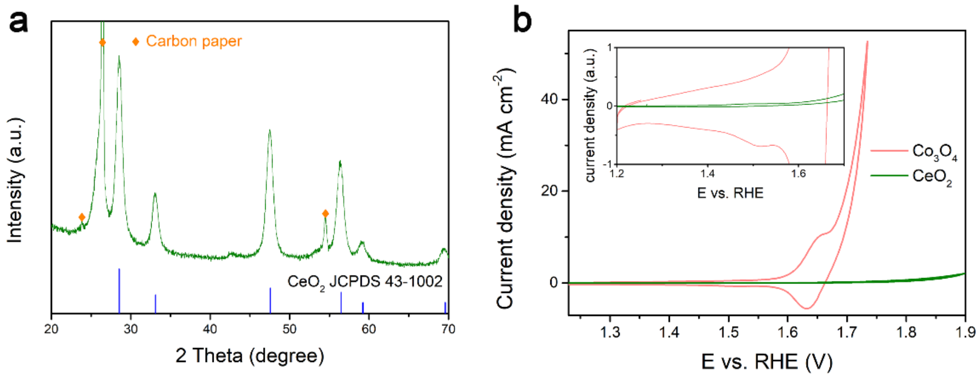

Supplementary Figure 9. (a) The XRD pattern for a CeO_2 sample on carbon paper (whose peaks are marked by orange dots) in comparison with the standard PXRD pattern (cubic, JCPDS 43-1002). (b) The CV curve of the CeO_2 sample compared to that of Co_3O_4 on carbon paper in 0.5 M H_2SO_4 solution. Inset in (b) is the enlarged image to see the redox features. Clearly, the CeO_2 sample show no obvious redox feature and very poor activity toward OER in acid.

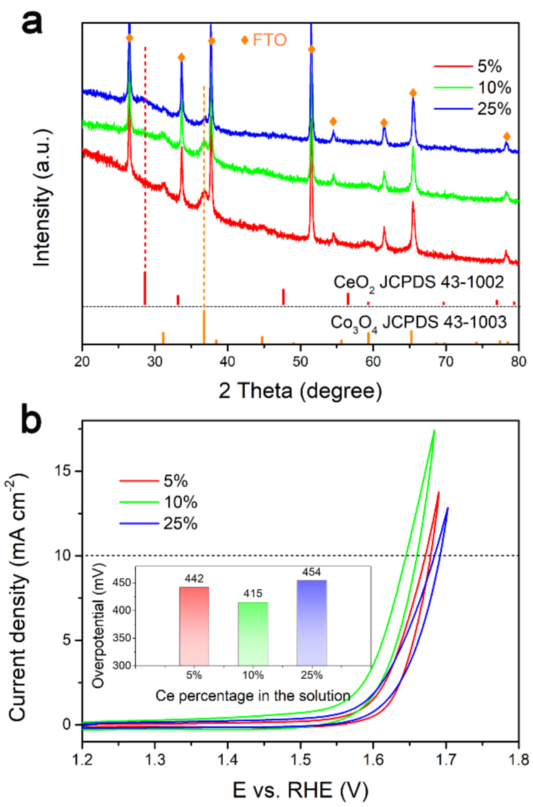

Supplementary Figure 10. (a) PXRD patterns of the series of Co_3O_4/CeO_2 catalysts on FTO electrodes prepared by introducing different nominal Ce metal contents (5, 10, and 25 mol%) during the electrodeposition process. (b) Comparisons of the acidic OER catalytic performances of the series of Co_3O_4/CeO_2 catalysts in 0.5 M H_2SO_4 solution, the inset shows the overpotential required to achieve a geometric catalytic current density of 10 mA cm^{-2} on each catalyst.

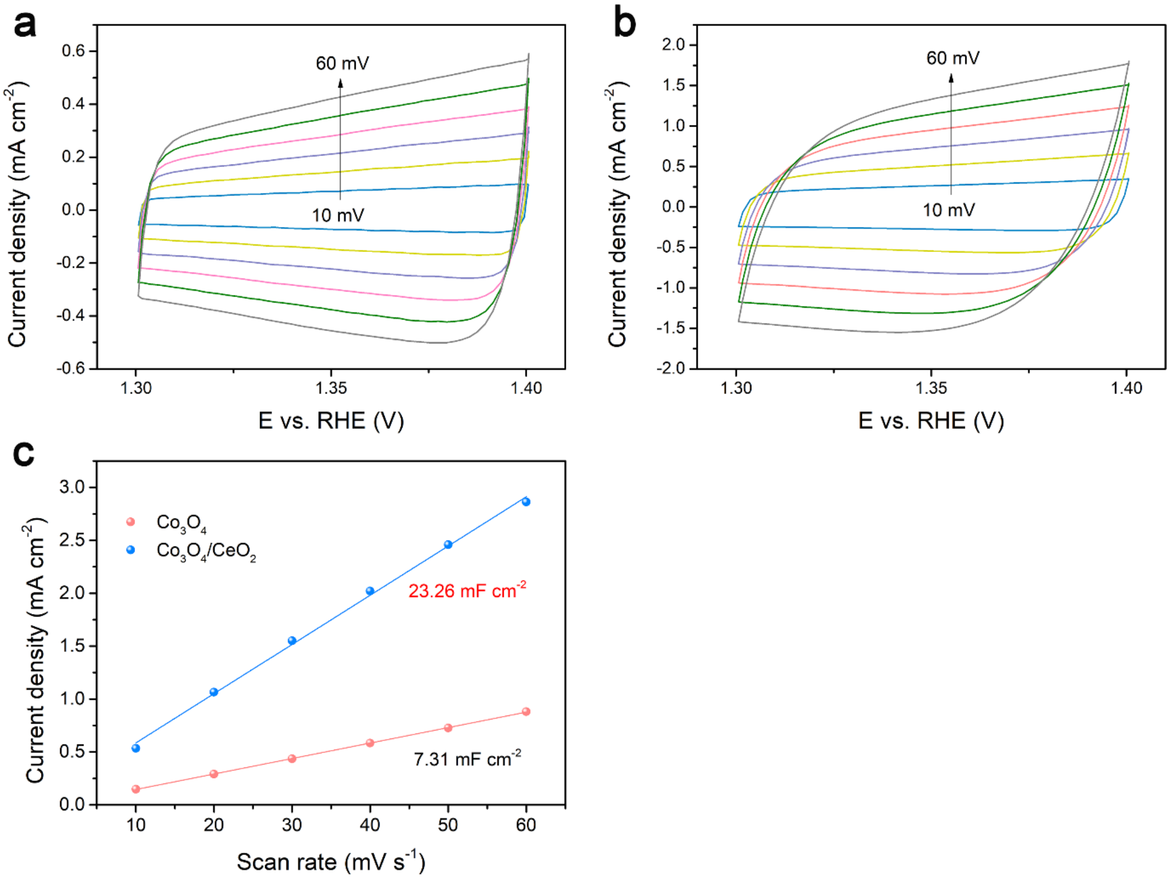

Supplementary Figure 11. CVs of (a) Co_3O_4 and (b) Co_3O_4/CeO_2 catalysts on FTO electrodes in 0.5 M H_2SO_4 solution recorded at different scan rates from 10 to 60 mV s^{-1} in pre-OER potential region. (c) C_{dl} values of both catalysts extracted from the plots of current density as a function of scan rate.

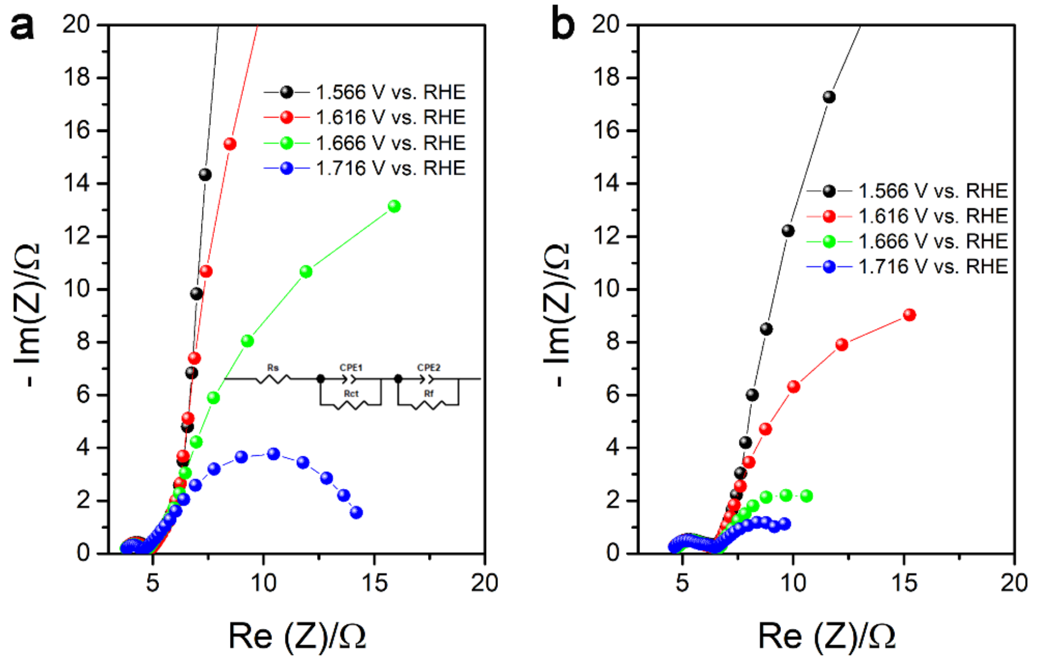

Supplementary Figure 12. The Nyquist plots of (a) Co_3O_4 and (b) Co_3O_4/CeO_2 catalysts on FTO electrodes recorded at different potentials in 0.5 M H_2SO_4 solution. The inset of (a) shows the Voigt circuit model. Since there are two semicircles in these Nyquist plots, the Voigt circuit is used as the equivalent circuit model for EIS fitting to extract the uncompensated solution resistance (R_s), the FTO/catalyst interface resistance (R_f), and the charge transfer resistance (R_{ct}) of the catalytic OER reaction. The R_{ct} values are summarized in Supplementary Table 2.

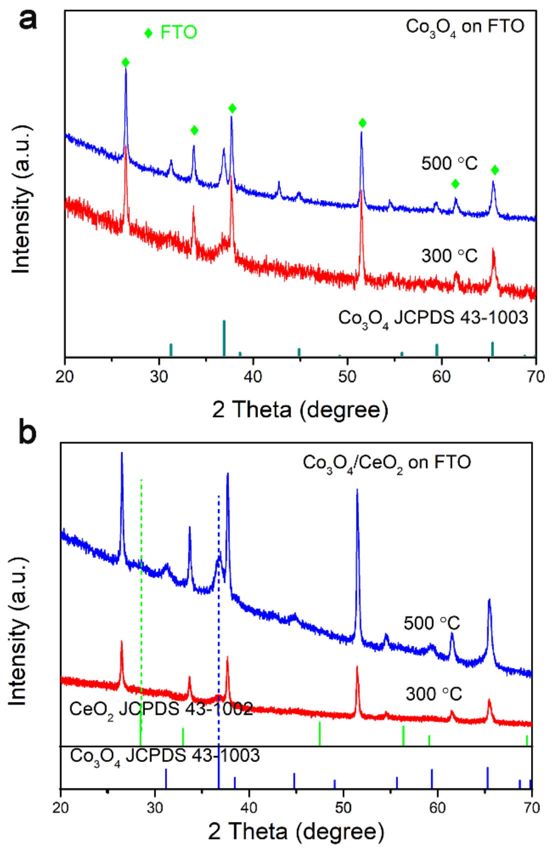

Supplementary Figure 13. PXRD patterns of the series of (a) Co_3O_4 and (b) Co_3O_4/CeO_2 catalysts on FTO electrodes prepared by annealing at different temperatures (300 vs. 500 °C) for 2 h. The samples annealed at 400 °C for 2 h are shown in Fig. 1e in the main text. The differences in diffraction peak widths clearly show that higher annealing temperatures resulted in higher degrees of crystallinity and larger crystalline domain sizes.

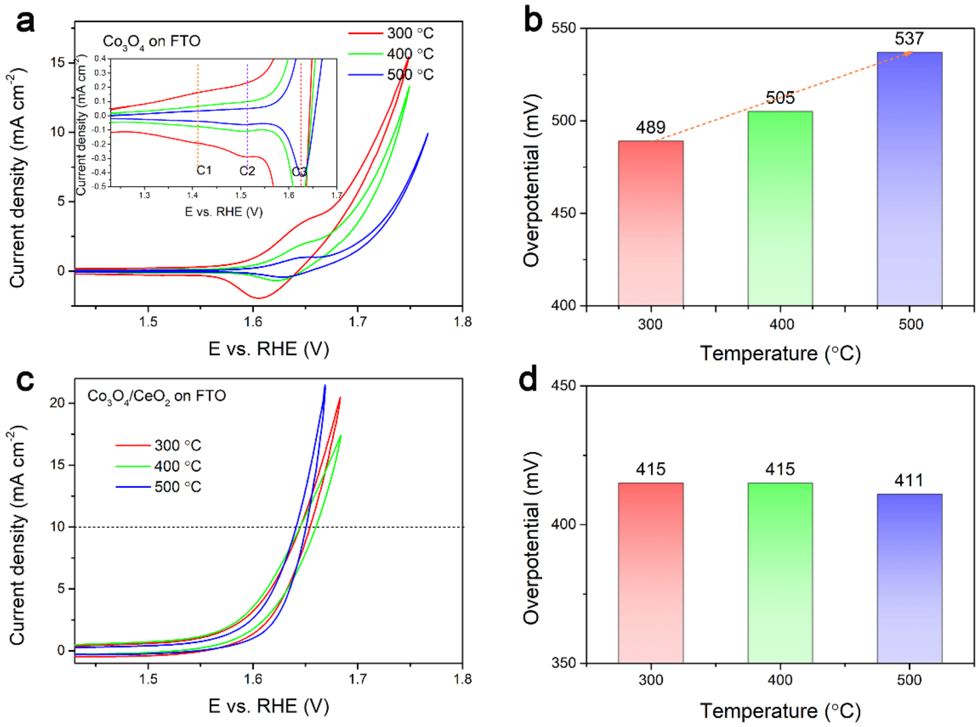

Supplementary Figure 14. Comparisons of the acidic OER catalytic performances of the series of (a) Co_3O_4 and (c) Co_3O_4/CeO_2 catalysts on FTO electrodes prepared by annealing at different temperatures (300, 400, or 500 °C) for 2 h. All CVs were recorded in 0.5 M H_2SO_4 solution. The inset of (a) shows the magnified CV curves of the Co_3O_4 catalysts that highlight the pre-OER redox features. The corresponding overpotentials required to achieve a geometric catalytic current density of 10 mA cm^{-2} on (b) Co_3O_4 and (d) Co_3O_4/CeO_2 catalysts annealed at different temperatures. Low temperature (less crystalline) Co_3O_4 catalyst had better catalytic performance; in contrast, the catalytic performance of Co_3O_4/CeO_2 nanocomposite catalysts was not sensitive to the annealing temperature (sample crystallinity).

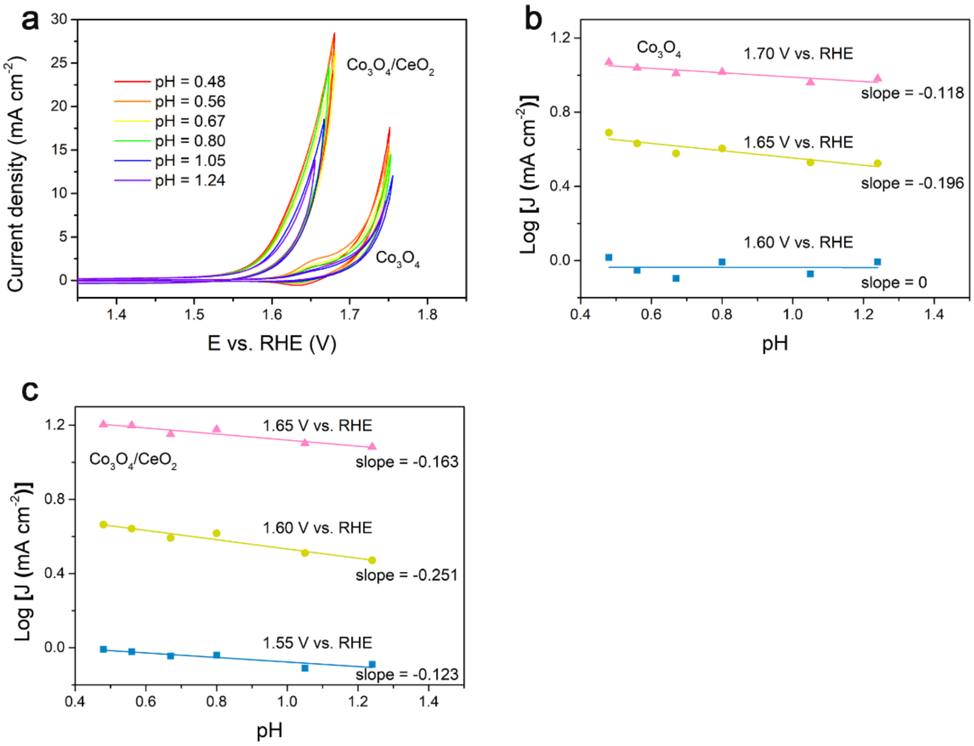

Supplementary Figure 15. (a) CV curves of both Co_3O_4 and Co_3O_4/CeO_2 catalysts on FTO electrodes recorded in H_2SO_4 solutions with different pH values of 0.48 (red), 0.56 (orange), 0.67 (yellow), 0.80 (green), 1.05 (blue), and 1.24 (purple). The logarithm of the catalytic current densities of (b) Co_3O_4 and (c) Co_3O_4/CeO_2 catalysts plotted against the solution pH at fixed potentials vs. RHE. The extracted reaction order with respect to pH is close to zero on the RHE scale for the acidic OER on both catalysts, indicating the catalytic reaction is less affected by the concentration of proton in the electrolyte.

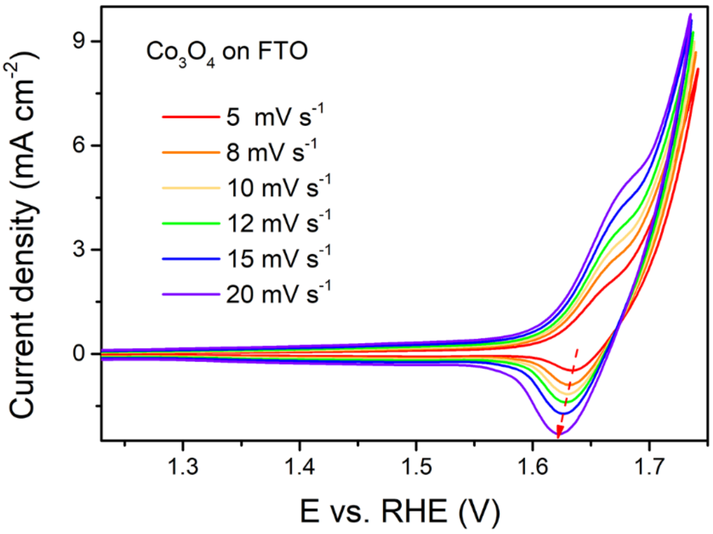

Supplementary Figure 16. CV curves of the Co_3O_4 catalyst on FTO electrode recorded at different scan rates in 0.5 M H_2SO_4 solution. Given the power law relationship between the cathodic peak current density (j_c) and the scan rate (v)[6,7]: $j_c = av^b$. The logarithm of the current densities of the three pre-OER cathodic peaks (denoted as C1, C2 and C3 in order of increasing potential) were plotted against the logarithm of the scan rates to extract the exponent b value of each redox process. In the two limiting cases: (i) $b = 0.5$ when it is a diffusion-controlled redox process; (ii) $b = 1$ when it is a perfectly non-diffusion-controlled capacitive behavior. The extracted exponent b values of C1, C2 and C3 peaks are 1.058 ± 0.022, 0.967 ± 0.007 and 1.122 ± 0.044, respectively, as shown in the inset of Fig. 3b in the main text. These exponent b values indicate all of these pre-OER redox features are surface capacitive processes.

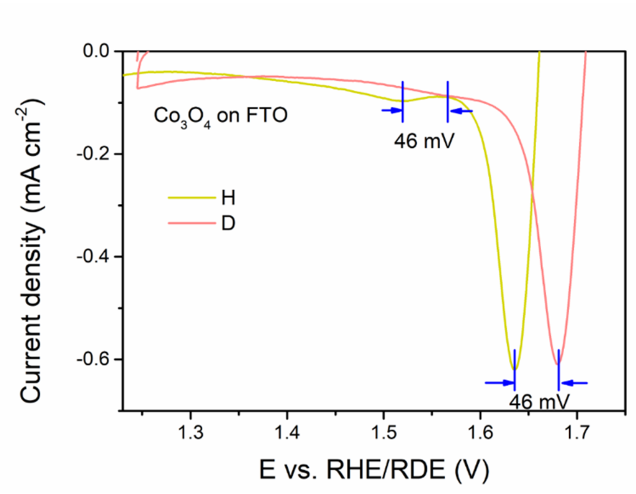

Supplementary Figure 17. Magnified CV curves of the Co_3O_4 catalyst on FTO electrode recorded in 0.5 M H_2SO_4 in H_2O solution on the RHE scale (yellow) vs. in 0.5 M D_2SO_4 in D_2O solution on the RDE scale (orange). A similar shift of ~46 mV was observed in the peak potentials of both C2 and C3 peaks in the protonic vs. deuteric solution, which is likely induced by the equilibrium isotope effect (EIE) associated with the redox processes of Co redox centers in Co_3O_4 sample[3].

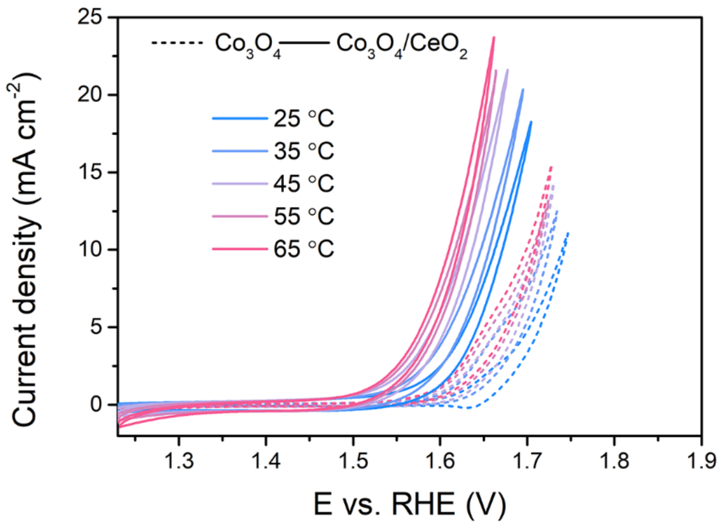

Supplementary Figure 18. CV curves of Co_3O_4 (dashed) and Co_3O_4/CeO_2 (solid) catalysts on FTO electrodes recorded in 0.5 M H_2SO_4 solution at different temperatures. These CV results were used to calculate the apparent activation energy (E_{app}) and the pre-exponential factor (A_{app}) for both catalysts, as shown in Figure 3f Supplementary Fig. 20a.

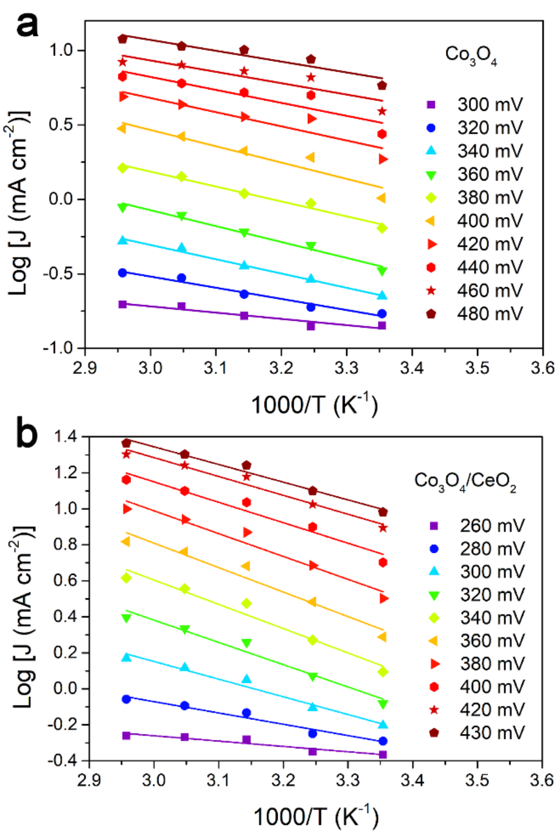

Supplementary Figure 19. The logarithm of the catalytic current density plotted against 1000 times the reciprocal of the temperature (in Kelvin) to extract the apparent activation energy (E_{app}) and the pre-exponential factor (A_{app}) of the acidic OER on (a) Co_3O_4 and (b) Co_3O_4/CeO_2 catalysts at fixed overpotentials using the Arrhenius plots (Equation 11 in Methods). The extracted E_{app} values are shown in Fig. 3f in the main text and the pre-exponential factors (A_{app}) are shown in Supplementary Fig. 20a.

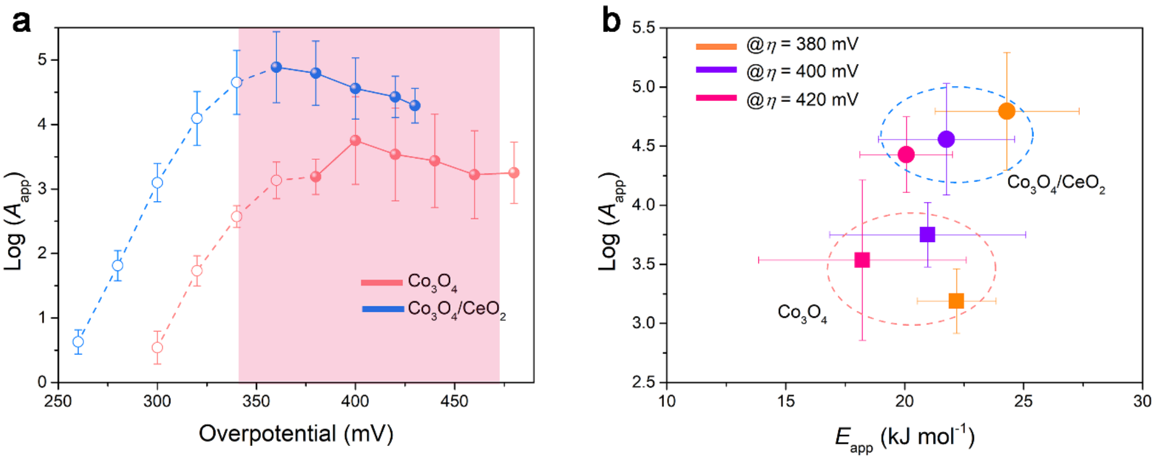

Supplementary Figure 20. (a) The logarithm of pre-exponential factor A_{app} derived from the intercept during the extraction of E_{app} for Co_3O_4 and Co_3O_4/CeO_2 catalysts at fixed overpotentials using the Arrhenius plots, as shown in Supplementary Fig. 19. (b) The logarithm of pre-exponential factor A_{app} plotted against E_{app} (at three representative overpotentials) to differentiate the major difference between Co_3O_4 and Co_3O_4/CeO_2. The error bar represents the standard error from fitting.

Generally speaking, E_{app} reflects the modification in enthalpy of activation and formation of intermediates, while A_{app} is often altered by changing the concentration of active sites or entropy of activation (the number of active intermediates that enter the rate determining step). A lower E_{app} and higher A_{app} will both lead to better catalytic performance[8,9]. Combining the smaller Tafel slope and higher intrinsic activity even after ECSA-normalization in Co_3O_4/CeO_2, it is quite likely that the introduction of CeO_2 alters the entropy of activation for Co_3O_4 since higher A_{app} values were consistently observed in Co_3O_4/CeO_2 nanocomposite. In contrast, the differences in the E_{app} values between Co_3O_4 and Co_3O_4/CeO_2 nanocomposite catalysts are less significant and consistent.

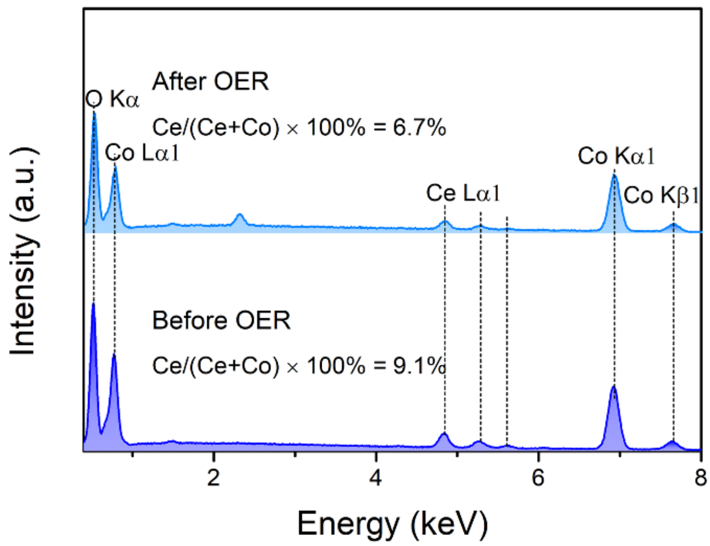

Supplementary Figure 21. SEM-EDS spectra of the Co_3O_4/CeO_2 catalyst before and after OER testing in 0.5 M H_2SO_4 solution.

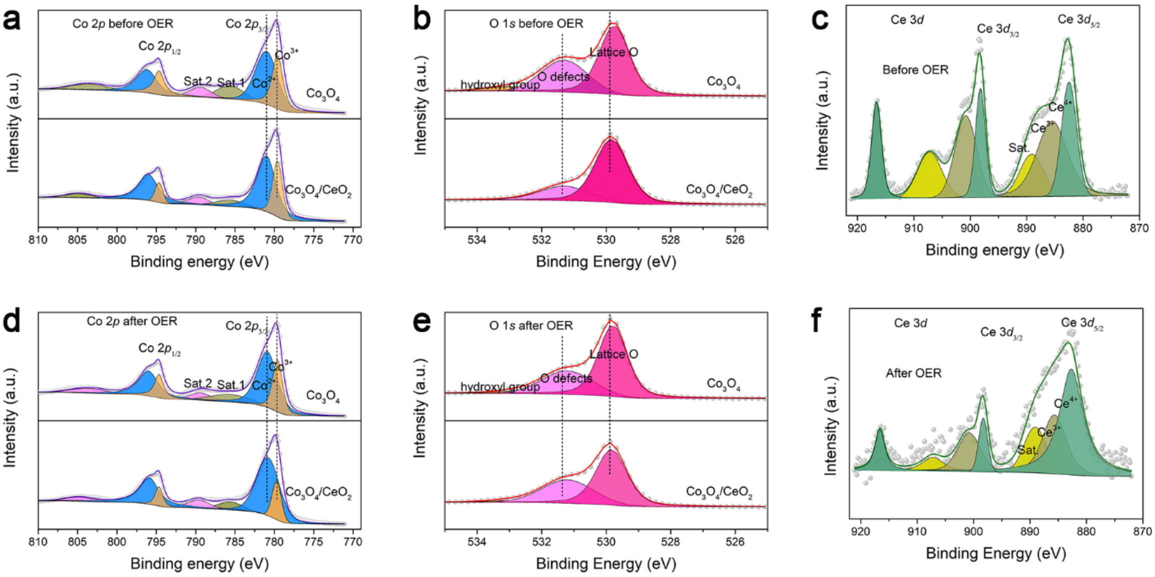

Supplementary Figure 22. High-resolution (a) Co 2*p*, (b) O 1*s* and (c) Ce 3*d* XPS spectra of Co_3O_4 and Co_3O_4/CeO_2 catalysts on FTO electrodes (a–c) before and (d–f) after OER testing in 0.5 M H_2SO_4 solution. Due to the spin-orbit coupling effect, the Co 2*p* signals are split into two dominant peaks, namely Co $2p_{3/2}$ and Co $2p_{1/2}$ (Supplementary Fig.22a,d). The Co $2p_{3/2}$ peak can be fitted into Co^{3+} and Co^{2+} components at the binding energy of 779.6 and 781.4 eV, respectively[10]. No obvious shift in the binding energies of the Co 2*p* signals is observed after the introduction of CeO_2. According to the XPS fitting results, Co_3O_4/CeO_2 exhibits a higher Co^{2+}/Co^{3+} ratio of 2.19 compared to Co_3O_4 (1.70), which is consistent with other reported Co_3O_4/CeO_2 nanocomposites[10-12]. The O 1*s* signal can be deconvoluted into three different oxygen species, namely the lattice oxygen that binds to metal (529.8 eV), the undercoordinated oxygen defect (531.4 eV), and the adsorbed hydroxyl or H_2O on the surface (533.0 eV) (Supplementary Fig. 22b,e). The Ce 3*d* signal of Co_3O_4/CeO_2 shows the co-existence of Ce^{3+} and Ce^{4+} (Supplementary Fig. 22c,f).

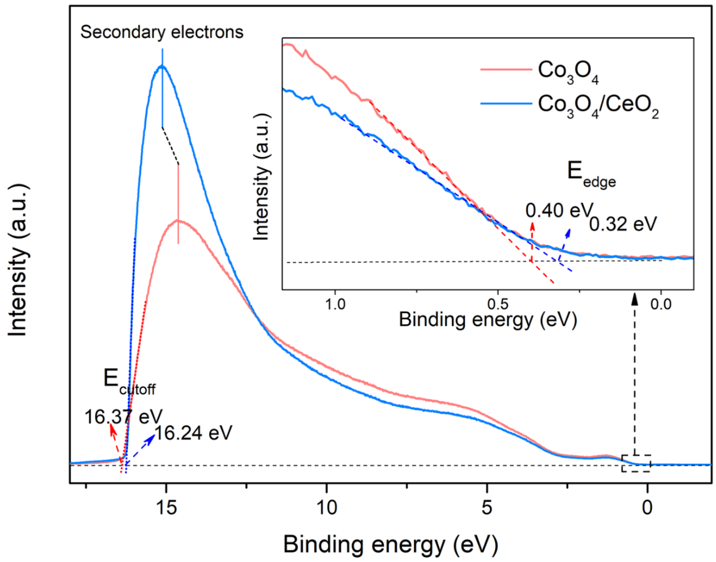

Supplementary Figure 23. The UPS spectra of Co_3O_4 and Co_3O_4/CeO_2 on FTO, the inset shows the enlarged spectra near the Fermi edge to highlight the difference at the valence band edge (E_{edge}).

Besides the variations in the intensity of UPS spectra, the differences between Co_3O_4 and Co_3O_4/CeO_2 are also observed in the cutoff energy (E_{cutoff}) for the secondary electrons and at the valence band edge (E_{edge}, reflecting the difference between the Fermi level and the valence band maximum). The work function ($\Phi = hv$ - E_{cutoff}[13,14], where hv is 21.22 eV for the excitation energy of the He I source) of Co_3O_4 and Co_3O_4/CeO_2 can be determined to be 4.85 and 4.98 eV, with the corresponding valence band energy [$E_{VB} = -(\Phi + E_{edge})$ vs. vacuum[13,14]] of –5.25 and –5.20 eV, respectively. The work function of CeO_2 was calculated to be 5.287 eV according to the literature[15]. Thus, the slightly modified work function of Co_3O_4/CeO_2 compared to Co_3O_4 can result from the charge redistribution between Co_3O_4 and CeO_2 to reach the equilibrium state. The charge redistribution across the Co_3O_4/CeO_2 nanocomposite interface can also be reflected by the higher average Co valence state and shorter Co-O bond distance from XAS results (Figure 4). The similar phenomenon has also been reported by Liu et al previously[12]. These results suggest possible electronic interactions in Co_3O_4/CeO_2 nanocomposite.

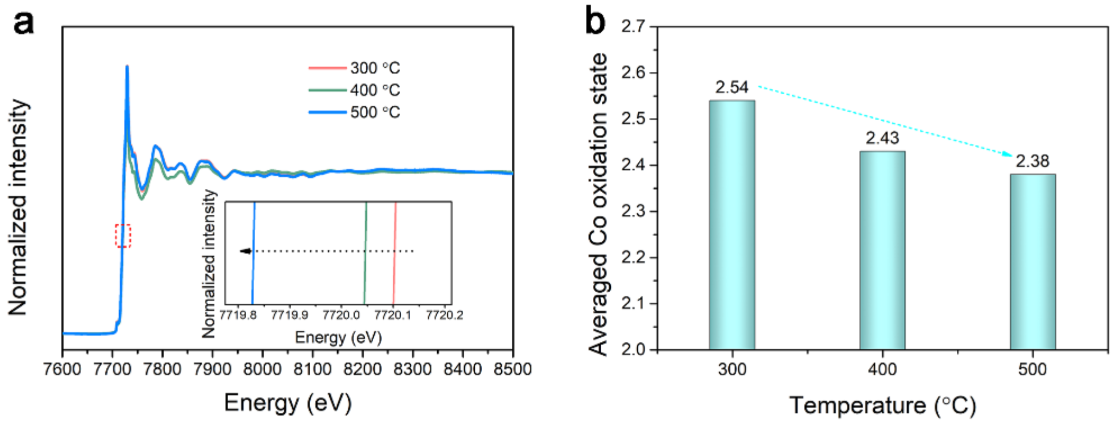

Supplementary Figure 24. Co K-edge XANES spectra of the as-synthesized Co_3O_4 catalysts annealed at 300, 400 and 500 °C. (b) Comparisons of the average Co valence states of the as-synthesized Co_3O_4 catalysts annealed at different temperatures.

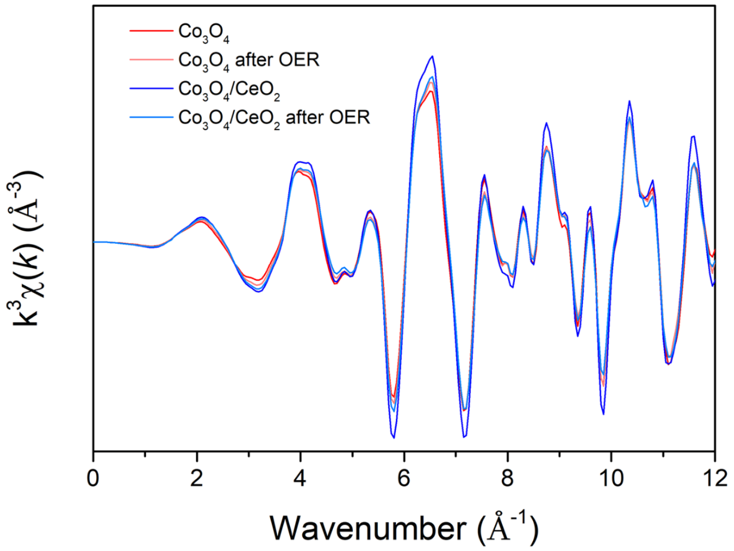

Supplementary Figure 25. $k^3\chi(k)$ functions of Co K-edge EXAFS spectra of Co_3O_4 and Co_3O_4/CeO_2 catalysts before and after OER testing in 0.5 M H_2SO_4 solution.

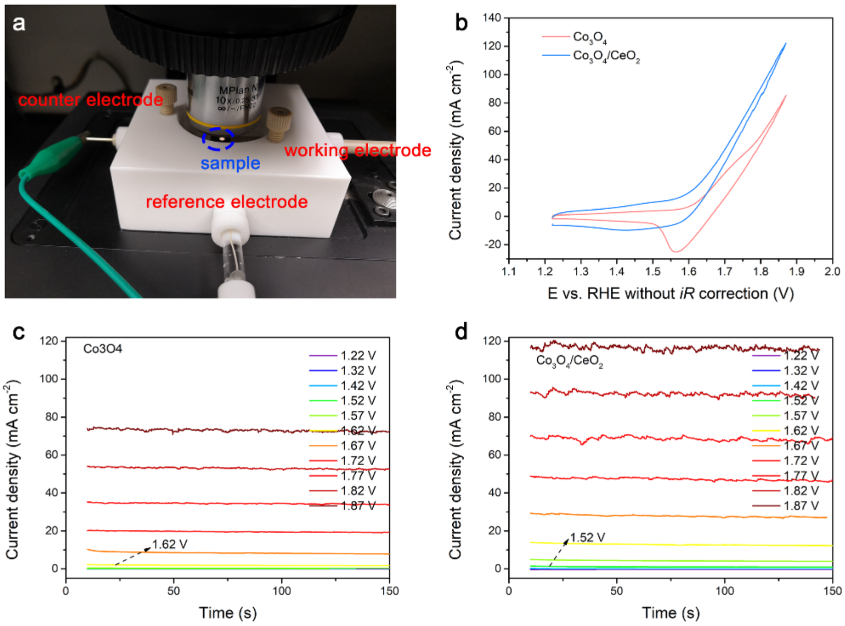

Supplementary Figure 26. (a) The experimental setup for in situ Raman measurements. (b) The CV curves of Co_3O_4 and Co_3O_4/CeO_2 catalysts on carbon paper electrodes collected at the scan rate of 20 mV s^{-1} in 0.5 M H_2SO_4 using the in situ Raman cell. (c,d) The chronoamperometry curves of (c) Co_3O_4 and (d) Co_3O_4/CeO_2 collected at various constant potentials (vs. RHE) during the in situ Raman measurements.

The Raman spectra were collected at various constant potentials without *iR* correction (increased from 1.22 V to 1.87 V vs. RHE and finally switched back to 1.22 V vs. RHE). Co_3O_4 showed the characteristic $Co^{III}Co^{IV} \leftrightarrow Co^{IV}Co^{IV}$ redox feature and the poorer OER performance than Co_3O_4/CeO_2 (Supplementary Figure 26b). The catalytic OER current on Co_3O_4 and Co_3O_4/CeO_2 was not obviously detected until 1.62 V and 1.52 V vs. RHE, respectively, suggesting the OER onset potential of Co_3O_4/CeO_2 was ~ 100 mV lower than that of Co_3O_4.

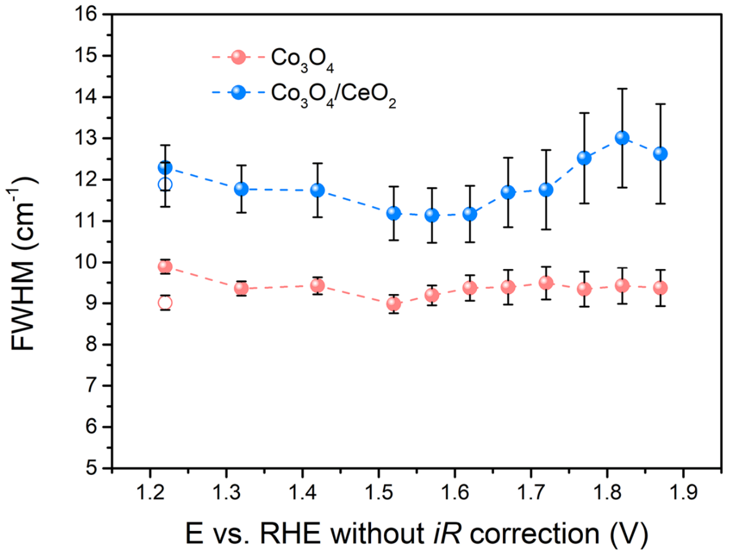

Supplementary Figure 27. The FWHM of the Raman A_{1g} peaks of Co_3O_4 and Co_3O_4/CeO_2 plotted against the applied potential show relatively small variations. The open symbols represent the data collected at 1.22 V after applying high potential (see Figure 5 in the main text for reference). Larger FWHM suggests smaller crystallites in Co_3O_4/CeO_2, which is consistent with the XRD (Supplementary Figure 5) and HRTEM (Figure 1c,d in the main text) results. The error bar represents the error from fitting.

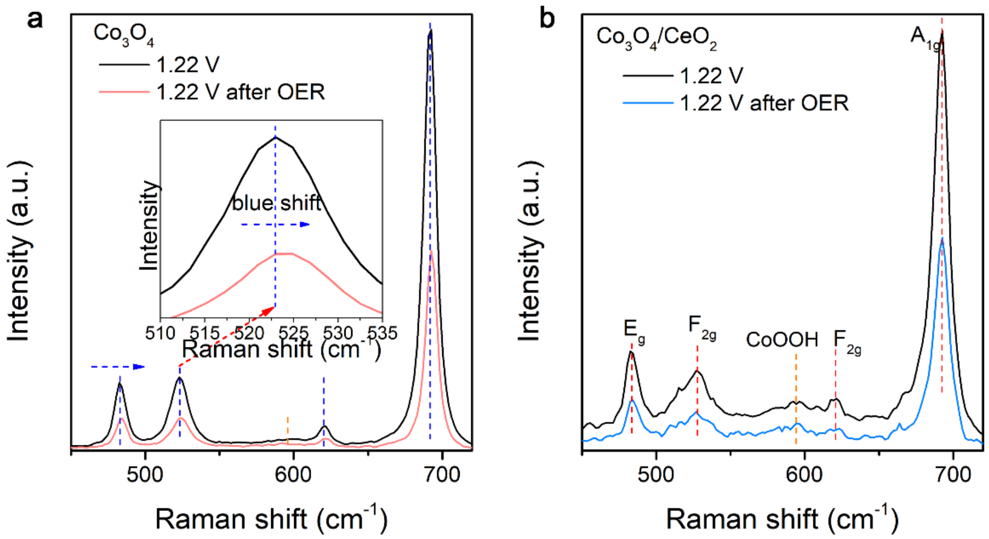

Supplementary Figure 28. The in situ Raman spectra of (a) Co_3O_4 and (b) Co_3O_4/CeO_2 collected at the 1.22 V vs. RHE before and after applying the higher potential sequence in the OER region up to 1.87 V vs. RHE (see Figure 5a in the main text for reference).

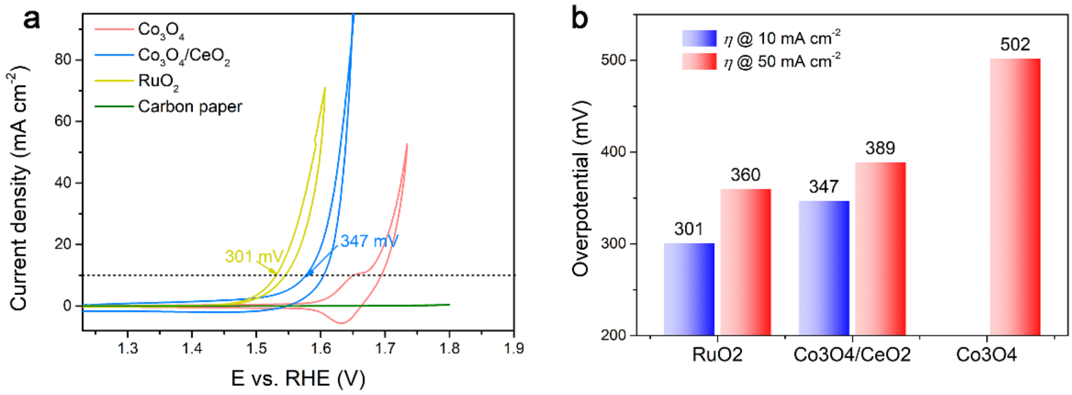

Supplementary Figure 29. (a) CV curves of Co_3O_4 and Co_3O_4/CeO_2 catalysts on carbon paper electrodes recorded in 0.5 M H_2SO_4 solution, in comparison with the bare carbon paper electrode and the benchmark RuO_2 catalyst on carbon paper electrode. (b) Comparisons of the overpotential required for each catalyst to reach a geometric current density of 10 and/or 50 mA cm^{-2} on carbon paper electrode. The PXRD patterns for the Co_3O_4 and Co_3O_4/CeO_2 catalysts on carbon paper electrodes are shown in Supplementary Fig. 5.

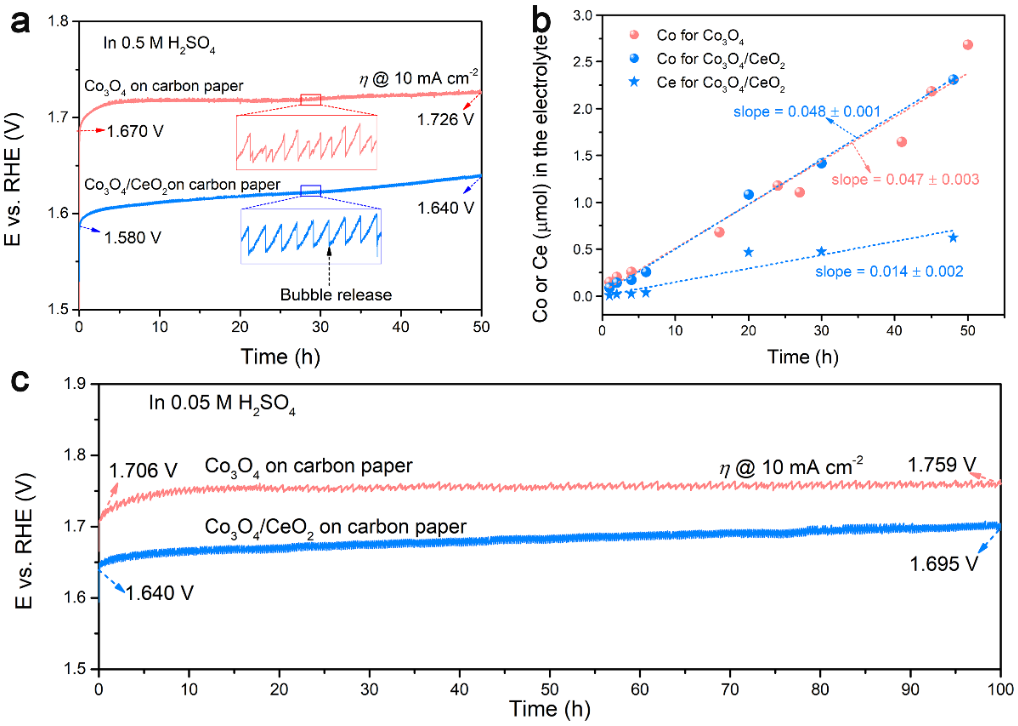

Supplementary Figure 30. Long-term chronopotentiometry tests of Co_3O_4 and Co_3O_4/CeO_2 catalysts on carbon paper electrodes. (a) Chronopotentiometry curves of both catalysts held at a constant geometric catalytic current density of 10 mA cm^{-2} in 0.5 M H_2SO_4 solution over 50 h. The insets show the magnified curves within the time window of 28 to 30 h revealing some small fluctuations of current electrode potentials likely due to the accumulation and release of oxygen gas bubbles. (b) The corresponding cobalt and/or cerium dissolution rates of both catalysts measured by ICP-MS during the chronopotentiometry tests in 0.5 M H_2SO_4 solution shown in (a). (c) Chronopotentiometry curves of both catalysts at 10 mA cm^{-2} in a less concentrated 0.05 M H_2SO_4 solution over 100 h.

Note that the high-performance carbon paper electrodes were used to better compare the stability of Co_3O_4 vs. Co_3O_4/CeO_2 using a chronopotentiometry method. The stability tests were first conducted in 0.5 M H_2SO_4 solution for 50 h at a constant current density of 10 mA cm^{-2} (Supplementary Fig. 30a). The potential increase over time on the two catalysts were comparable (a 56 and 60 mV increase over 50 h in the case of Co_3O_4 and Co_3O_4/CeO_2, respectively). The dissolution rates of metal ions were monitored by collecting the electrolyte solution at different time points and performing ICP-MS analysis, as shown in Supplementary Fig. 30b. The cobalt dissolution rates of both catalysts followed almost the same linear trend (~ 0.047 and 0.048 μmol h^{-1} in the case of Co_3O_4 and Co_3O_4/CeO_2, respectively), suggesting the dissolution rate of Co in both catalysts on carbon paper are ~ 50 ng min^{-1}, which is a little lower than the 100 ng min^{-1} reported for Co_3O_4 on FTO under the same testing condition[16]. The stability tests were further performed in a less concentrated 0.05 M H_2SO_4 solution for a longer time period of 100 h, again showing very similar rates of potential increase over time on the two catalysts (53 and 55 mV

increase over 100 h in the case of Co_3O_4 and Co_3O_4/CeO_2, respectively) (Supplementary Fig. 30c).

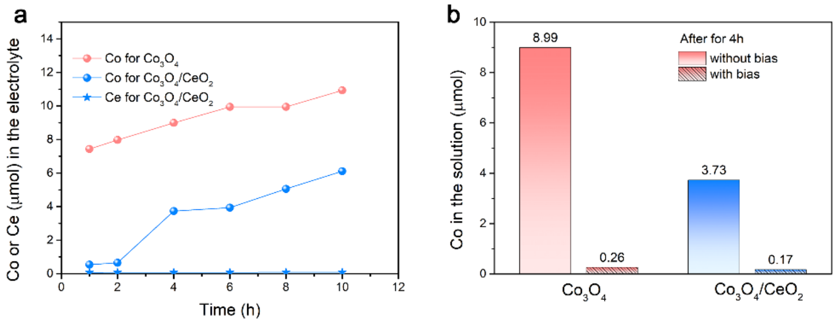

Supplementary Figure 31. (a) The metal dissolution rates of Co_3O_4 and Co_3O_4/CeO_2 catalysts without a bias. (b) The comparison of the amount of Co ions in the electrolyte solutions leached from both Co_3O_4 and Co_3O_4/CeO_2 catalysts after 4 h with or without a bias.

Supplementary Table 1. The bulk and surface percentage Ce metal contents in the Co_3O_4/CeO_2 catalyst [defined as Ce/(Ce + Co) × 100%] determined by SEM-EDS and XPS, respectively, before and after OER testing in 0.5 M H_2SO_4 solution.

	SEM-EDS	XPS
Before OER	9.1	6.6
After OER	6.7	2.0

Supplementary Table 2. The extracted R_{ct} values of the catalytic OER on both Co_3O_4 and Co_3O_4/CeO_2 catalysts on FTO electrodes at different potentials (V vs. RHE) in 0.5 M H_2SO_4 solution.

	Co_3O_4 (Ω)	Co_3O_4/CeO_2 (Ω)
1.566	1100	129.0
1.616	231.9	25.2
1.666	52.9	6.8
1.716	10.5	4.3

Supplementary Table 3. Comparisons of the catalytic performances of Co_3O_4/CeO_2 in this work with other reported earth-abundant metal oxide-based electrocatalysts in acidic OER.

Catalyst	Electrolyte	Overpotential (Current Density)	Reference
Ba[Co-POM] with carbon paste	1 M H_2SO_4	189 mV (1 mA cm^{-2})	*Nat. Chem.* 2018, *30*, 24–30.
$CoFeBbO_x$ on Pt/Ti electrode[a]	1 M H_2SO_4	700 mV (500 mA cm^{-2})	*Nat. Catal.* 2019, 2, 457–465
γ-MnO_2 on carbon paper	1 M H_2SO_4	428 mV (10 mA cm^{-2})	*Angew. Chem. Int. Ed.* 2019, *58*, 1–6.
$Ni_{0.5}Mn_{0.5}Sb_{1.7}O_y$ on ATO	1 M H_2SO_4	672 mV (10 mA cm^{-2})	*Energy Environ. Sci.*, 2017, *10*, 2103–2108.
$Mn_xSb_{1-x}O_2$ on Si	1 M H_2SO_4	580 mV (50 mA cm^{-2})	*ACS Catal.* 2018, *8*, 10938−10948.
F-doped $Cu_{1.5}Mn_{1.5}O_4$ on glassy carbon	0.5 M H_2SO_4	330 mV (10 mA cm^{-2})	*Sci. Rep.* 2016, *6*, 28367.
Fe-TiO_x on Ti foam	0.5 M H_2SO_4	261 mV (1 mA cm^{-2})	*Nano Energy* 2018, *45*, 118–126.
Ag-doped Co_3O_4 on glassy carbon	0.5 M H_2SO_4	470 mV (10 mA cm^{-2})	*J. Mater. Chem. A* 2018, *6*, 5678–5686.
Co_3O_4 on FTO	0.5 M H_2SO_4	570 mV (10 mA cm^{-2})	*Chem. Mater.* 2017, *29*, 950−957.
Co_3O_4/CeO_2 on FTO	0.5 M H_2SO_4	423 mV (10 mA cm^{-2})	This work
Co_3O_4/CeO_2 on carbon paper	0.5 M H_2SO_4	347 mV (10 mA cm^{-2})	This work
Fe_2O_3 on Ti foil[b]	0.5 M H_2SO_4	650 mV (10 mA cm^{-2})	*J. Catal.* 2018, *365*, 29–35.
Co-doped Fe_2O_3 on Ti foil[b]	0.5 M H_2SO_4	650 mV (10 mA cm^{-2})	*Chem. Commun.* 2019, *55*, 5017–5020.
Hetero-N-Coordinated Co on carbon paper[c]	0.5 M H_2SO_4	256 mV (10 mA cm^{-2})	*ACS Energy Lett. 2019, 4, 1816−1822*
N-doped WC on carbon fiber paper[c]	0.5 M H_2SO_4	470 mV (60 mA cm^{-2})	*Nat. Commun.* 2018, 9, 924

[a] tested at the 80 °C. [b] Fe_2O_3 is less stable compared to Co_3O_4. [c] Those catalysts are not oxide-based materials.

References Cited in the Supplementary Information

1 Costentin, C., Porter, T. R. & Savéant, J.-M. Conduction and Reactivity in Heterogeneous-Molecular Catalysis: New Insights in Water Oxidation Catalysis by Phosphate Cobalt Oxide Films. *J. Am. Chem. Soc.* **138**, 5615-5622, (2016).

2 Zhang, M., de Respinis, M. & Frei, H. Time-resolved observations of water oxidation intermediates on a cobalt oxide nanoparticle catalyst. *Nat. Chem.* **6**, 362-367, (2014).

3 Pasquini, C. *et al.* H/D Isotope Effects Reveal Factors Controlling Catalytic Activity in Co-Based Oxides for Water Oxidation. *J. Am. Chem. Soc.* **141**, 2938-2948, (2019).

4 Bergmann, A. *et al.* Unified structural motifs of the catalytically active state of Co(oxyhydr)oxides during the electrochemical oxygen evolution reaction. *Nat. Catal.* **1**, 711-719, (2018).

5 Moysiadou, A., Lee, S., Hsu, C.-S., Chen, H. M. & Hu, X. Mechanism of Oxygen Evolution Catalyzed by Cobalt Oxyhydroxide: Cobalt Superoxide Species as a Key Intermediate and Dioxygen Release as a Rate-Determining Step. *J. Am. Chem. Soc.* **142**, 11901-11914, (2020).

6 Brezesinski, T., Wang, J., Polleux, J., Dunn, B. & Tolbert, S. H. Templated Nanocrystal-Based Porous TiO_2 Films for Next-Generation Electrochemical Capacitors. *J. Am. Chem. Soc.* **131**, 1802-1809, (2009).

7 Xiao, J. *et al.* Rational Design of a P2-Type Spherical Layered Oxide Cathode for High-Performance Sodium-Ion Batteries. *ACS Cent. Sci.* **5**, 1937-1945, (2019).

8 Shinagawa, T. & Takanabe, K. New Insight into the Hydrogen Evolution Reaction under Buffered Near-Neutral pH Conditions: Enthalpy and Entropy of Activation. *J. Phys. Chem. C* **120**, 24187-24196, (2016).

9 Duan, Y. *et al.* Revealing the Impact of Electrolyte Composition for Co-Based Water Oxidation Catalysts by the Study of Reaction Kinetics Parameters. *ACS Catal.* **10**, 4160-4170, (2020).

10 Qiu, B. *et al.* CeO_2-Induced Interfacial Co^{2+} Octahedral Sites and Oxygen Vacancies for Water Oxidation. *ACS Catal.* **9**, 6484-6490, (2019).

11 Kim, J.-H. *et al.* Enhanced Activity Promoted by CeO_x on a CoO_x Electrocatalyst for the Oxygen Evolution Reaction. *ACS Catal.* **8**, 4257-4265, (2018).

12 Liu, Y. *et al.* 2D Electron Gas and Oxygen Vacancy Induced High Oxygen Evolution Performances for Advanced Co_3O_4/CeO_2 Nanohybrids. *Adv. Mater.* **31**, 1900062, (2019).

13 Liu, F. *et al.* Direct Z-Scheme Hetero-phase Junction of Black/Red Phosphorus for Photocatalytic Water Splitting. *Angew. Chem. Int. Ed.* **58**, 11791-11795, (2019).

14 Liu, W. *et al.* Single-Site Active Cobalt-Based Photocatalyst with a Long Carrier Lifetime for Spontaneous Overall Water Splitting. *Angew. Chem. Int. Ed.* **56**, 9312-9317, (2017).

15 Goldsby, J. Basic Elastic Properties Predictions of Cubic Cerium Oxide Using First-Principles Methods. *J. Ceram.* **2013**, 1-4, (2013).

16 Mondschein, J. S. *et al.* Crystalline Cobalt Oxide Films for Sustained Electrocatalytic Oxygen Evolution under Strongly Acidic Conditions. *Chem. Mater.* **29**, 950-957, (2017).
